# Evaluation of the Effectiveness of Teduglutide Treatment in Patients with Short Bowel Syndrome in Slovakia—Multicenter Real-World Study

**DOI:** 10.3390/jcm13051238

**Published:** 2024-02-22

**Authors:** Laura Gombošová, Martin Suchanský, Juraj Krivuš, Jarmila Hornová, Zuzana Havlíčeková, Andrea Fojtová, Barbora Norek, Iveta Valachová, Jana Šprláková, Jakub Gazda, Martina Ondrušová

**Affiliations:** 1 2nd Internal Clinic, University Hospital of L. Pasteur and Faculty of Medicine University of Pavol Jozef Šafárik Košice, Tr. SNP 1, 04011 Košice, Slovakia; jakub.gazda@upjs.sk; 2 PharmIn Ltd., Karadžičova 16, 82108 Bratislava, Slovakia; martin.suchansky@pharmin.sk; 3 1st Internal Clinic, University Hospital and Jessenius Faculty of Medicine Comenius University, Kollárova 2, 03659 Martin, Slovakia; jurajkrivus@gmail.com; 4 Department of Pediatrics, Faculty of Medicine Comenius University and National Institute of Children’s Diseases, Limbová 1, 83340 Bratislava, Slovakia; jarmila.hornova@gmail.com; 5 Department of Paediatrics, University Hospital Martin and Jessenius Faculty of Medicine Comenius University, Kollárova 2, 03601 Martin, Slovakia; zhavlicekova@gmail.com; 6 Gastroenterology Clinic, Slovak Medical University and Bratislava University Hospital, Antolská 11, 85107 Bratislava, Slovakia; andreafojtova@gmail.com (A.F.); barbora.norek@gmail.com (B.N.); 7 2nd Children Clinic, Slovak Medical University and Children's Faculty Hospital, Nám L. Svobodu 4, 97409 Banská Bystrica, Slovakia; iveta.valachova@dfnbb.sk; 8 Gastroenterology and Hepatology Department, Children’s Faculty Hospital, Tr. SNP 1, 04011 Košice, Slovakia; jana.sprlakova@dfnke.sk; 9 Faculty of Public Health, Slovak Medical University, 83303 Bratislava, Slovakia

**Keywords:** short bowel syndrome, teduglutide, HPN, weaning off, adults, children

## Abstract

(1) **Background:** We present the first real-world-data study on teduglutide-treated SBS patients in the Slovak Republic and the first study to enable the comparison of the effects of teduglutide treatment between the adult and pediatric populations. (2) **Methods:** This was a non-interventional retrospective cohort study of adult and pediatric SBS patients treated with teduglutide. Primary and secondary endpoints were the results of teduglutide use at 12 weeks and 6 months after the initiation of treatment, compared to baseline. (3) **Results:** Teduglutide treatment led to a statistically significant reduction in the volume of intravenous hydration, HPN caloric intake, HPN and intravenous hydration applications per week and to increased urine output in adult patients. The results in the pediatric population were similar, but not statistically significant. A complete weaning off HPN was achieved in 57.14% of all patients (50.00% of children; 62.50% of adults) after a median of 0.99 years of teduglutide treatment (1.07 and 0.98 years for children and adults, respectively). (4) **Conclusions:** Teduglutide treatment in SBS patients leads to considerable reduction in or even weaning off PN in both pediatric and adult patients.

## 1. Introduction

Short bowel syndrome (SBS) is a rare but life-threatening condition. It either arises due to the loss of function of the small intestine, or due to an extensive surgical resection leaving less than 200 cm of small intestine [1,2] or is congenitally present in newborns. SBS is the most frequent mechanism of intestinal failure (IF) [2]. Patients with SBS suffer from serious malabsorption and are unable to maintain a balance of macro- and micro-nutrients, electrolytes, and water through the gastrointestinal tract [3].

The most common underlying diseases in adult SBS patients are Crohn’s disease (CD), mesenteric ischemia (MI), and malignancy [4,5]. In children, the causes of SBS can arise in the prenatal period, with examples including abdominal wall defect, volvulus, atresia, or extensive agangliosis. The most common causes of SBS in the neonatal period are necrotizing enterocolitis, complicated meconium ileus, abdominal wall defects, intestinal atresia, or volvulus [6]. In older children, the most common causes are trauma, volvulus, and CD [7].

There are three degrees of SBS: type I, the most common type, is acute and may require short-term parenteral nutrition (PN); type II, the prolonged acute form, usually requires several weeks or months on PN; and type III, the chronic form, may be either reversible (months or years on PN) or irreversible (life-long PN) [8].

Management of SBS patients requires a multidisciplinary approach, including surgical, pharmacological, and dietary solutions [3]. Patients with intestinal failure (IF) usually require long-term PN or home parenteral nutrition (HPN) [8,9]. However, the use of long-term PN or HPN has its own complications, such as high cost, impaired quality of life, and high morbidity and mortality [10,11]. The most common catheter-related medical complications are catheter infection and sepsis, catheter occlusions, central vein thrombosis, tunnel infection, air embolism, and endocarditis [12,13]. Metabolic complications include hepatic steatosis; liver failure; oxalate nephropathy; gastroparesis and intestinal hypoplasia; cholecystolithiasis; metabolic bone disease; anemia; disorders of glucose, lipid, water, ion, and vitamin metabolism; manganese toxicity, and others [12,13,14].

The frequency of medical complications in HPN varies. A study of data from the USA reported a prevalence of metabolic complications in HPN patients of 0.12–0.61 episodes per catheter year [15]. A British study reported an overall rate of infection of 0.39 per 1000 catheter days [16]. About one-third of new HPN patients experience a treatment-related complication within the first 90 days on HPN; those complications may be infectious (56.8%), technical (25%), or metabolic (18.2%) [17]. A study from 1995 found that the probability of one-year survival for patients without malignancies patients with HPN dependency was 91%, whereas that of five-year survival was 62% [18]. According to Dibb et al. [19], the one-year survival probability for patients with long-term HPN is 93%, while the five-, ten-, and twenty-year survival prognoses are 71%, 59%, and 28%, respectively. A recent study from Canada found a five-year survival rate of >80% in SBS patients without malignancies [20].

The actual incidence and prevalence of SBS have not been fully clarified, mostly because of under-reporting and the absence of reliable patient databases [21,22,23], but available data show us that both the incidence and prevalence of SBS have increased in recent decades [3,24]. In Europe, the incidence is estimated at two to three cases per million and the prevalence is estimated at four cases per million [3,21,25]. The best estimate is based on the number of patients receiving long-term PN, given that a significant percentage of patients on PN have SBS (35%) [21,26]. However, patients no longer being treated with PN are not included, and thus the number of SBS patients is underestimated by this method [3,27].

It is not possible to determine the exact prevalence and incidence of short bowel syndrome in the Slovak population due to the lack of specialized registers and databases for this syndrome. The only complex source of information is the Slovak Register of Patients on Home Parenteral Nutrition [28]. According to this register, there were n = 76 adult SBS patients as of 16 November 2022. According to the register and to an anonymized retrospective quantitative survey among physicians, there were n = 21 pediatric SBS patients in the Slovak Republic as of 16 November 2023 [29].

The main goal of recent approaches in the management of SBS patients is to enhance the absorption capacity of the rest of the small intestine, and in this way to achieve reduced HPN dependency or a complete weaning off HPN [8]. For this purpose, non-transplant surgical procedures can be used to improve the function and motility of the intestine, to increase the absorption surface of the mucous membrane, and to prolong intestinal transit time.

Pharmacologic therapy for SBS is a rapidly expanding area of investigation. Teduglutide (REVESTIVE^®^), a glucagon-like peptide-2 analogue, increases intestinal absorption by increasing blood flow to and from the gut, reducing the speed at which food passes through, and reducing acid secretions in the stomach, as these can interfere with absorption in the intestine. Teduglutide also has the advantage of lasting longer than GLP-2 in the body [30]. Clinical trials showed improved intestinal absorption and reduced HPN requirements in adult and pediatric patients [8,30,31,32,33].

Here, we present a non-interventional retrospective cohort study evaluating basic characteristics of the Slovak SBS patient population (children and adults) on teduglutide treatment (n = 16) and the effect of teduglutide on selected health indicators.

We performed separate analyses of children and adults. This separation is based on the different nutritional requirements of pediatric patients and the possible effect of children’s physiological development on individual parameters [34,35].

## 2. Materials and Methods

This was a non-interventional retrospective cohort study that evaluated the chronological medical records of patients followed long-term by their specialists (gastroenterology/hepatology and pediatrics facilities in the Slovak Republic). The data were collected from the electronic and physical medical records into a standardized case-report form. The last data update was done on 4 November 2023. The study population consisted of a general (non-selective) sample, i.e., all teduglutide-treated SBS patients in outpatient clinics in Slovakia who met the eligibility criteria (n = 16). This was a case-crossover study with a self-controlled design, wherein each patient acts as his or her own control.

### 2.1. Participants and Setting

Data on all pediatric and adult patients diagnosed with SBS whose first teduglutide treatment (index date) was between 9 September 2020 and 16 November 2022 and who completed at least 6 months of teduglutide treatment were collected. Patients were treated with the recommended dose 0.05 mg/kg/day of teduglutide (Revestive^®^, Shire Pharmaceuticals Ireland Limited, Dublin, Ireland and Takeda Pharmaceuticals International AG Ireland Branch, Dublin, Ireland). As teduglutide was not included in the list of reimbursed drugs in Slovakia at that time, approval by the health insurance company was required for reimbursement when the treatment was indicated according to the Summary of Product Characteristics (SmPC).

Patient characteristics and measurements were collected at three time points: at baseline, defined as the last available medical check-up prior to the index date, and at two follow-up time points, 12 weeks and 6 months after the index date. The study also included descriptive data from the time of SBS diagnosis. Each participating center granted permission to use medical-chart data in this noninterventional case-crossover study. There was no direct contact with the subjects or primary collection of individual data. No patient identifiers were included, and the protocol did not interfere with physicians’ decisions on patient care management. The results are summarized in tabular form, omitting subject identification, and the study was approved by the Ethics Committee of University Hospital of L. Pasteur, Kosice, Slovakia (Ethics Committee No. consent 2023/EK/08039).

### 2.2. Primary and Secondary Outcomes

**Figure 1:**
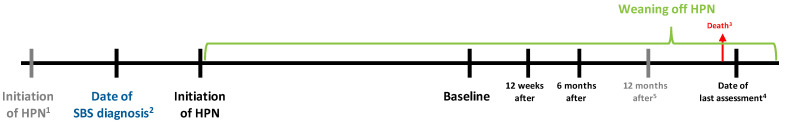
Timeline of the study. ^1^ In one adult patient, HPN was initiated before the SBS diagnosis. ^2^ In 5 patients, HPN was initiated at the day the SBS diagnosis was confirmed. ^3^ One patient (a child) died before the end of the study (after 410 days of teduglutide treatment) due to fulminant sepsis. ^4^ 6 patients were still HPN-dependent at the end of the study. ^5^ The “12-month” time point was calculated for the purpose of statistical evaluation of HPN status. Other parameters of the study were not included in the analysis.

The primary objectives of this study were to characterize the profiles of all patients with SBS who were treated with teduglutide in Slovakia at defined time points and to describe the results of teduglutide use at the 12-week and 6-month follow-ups. The primary outcome of the study was the gradual weaning off HPN. This parameter was referred to as “weaning-off status” (referring to weaning off any form of HPN, including both nutrition and hydration) at two time points: 12 weeks and 6 months. However, larger proportions of patients who had weaned off teduglutide were observed after a longer period than originally intended for the analyses. For this reason, an expanded graded primary end point was introduced to analyze the weaning-off status of these patients at a 12-month follow-up and at the end of the study (date of the last assessment, 4 November 2023). The calculation of the “12-month” time point was based on the known date of the first and the last HPN administration. Other evaluated parameters were not monitored for these two additionally exploratory time points because the “12-month” and the “date of the last assessment” time points were introduced during the study and because the patients had started the teduglutide treatment at different times (each patient had a floating treatment period). The timeline of the study is depicted in Figure 1.

In addition to weaning-off status, the effect of the gradual reduction in the HPN-dependence of patients can be indirectly observed through the other monitored quantitative parameters (intravenous hydration volume per week, intravenous hydration applications per week, HPN caloric intake per week, HPN caloric intake applications per week, BMI, urine output per day and number of stools per day), which we can consider as secondary outcomes.

The secondary objectives of this study were to evaluate the differences in individual changes in the evaluated parameters between baseline and follow-up. The peroral caloric intake per day (including nutritional products) could not be quantified because only data available from the patients were rounded to thousands of kcal were. Thus, for the purpose of this study, this parameter was categorized according to the standard peroral income of an average person of a similar age: double, normal, two-thirds, half, and one-third normal intake. We specified the oral intake in kcal. In adults, hyperphagia was observed. We observed increased energy intake, which is in line with recommendations for patients with SBS.

Energy requirements could be met by supplying 1.4 times the resting energy expenditure (REE), or about 30 kcal/kg/d. Many stable patients on parenteral nutrition are satisfactorily maintained on 20–35 kcal total energy per kg per day [36]. Hyperphagia is defined as oral intake 1.5 times greater than the patient’s resting energy expenditure. Based on clinical experience, spontaneous hyperphagia is reported in 70% of adult patients with SBS [37].

Primary and secondary outcomes were described for all patients except for one adult patient, who started teduglutide treatment 1.8 years after the end of PN due to the loss of central venous access sites. The patient survived due to significantly increased p.o. energy intake, but diarrhea and weight loss persisted, which led to poor quality of life. This patient was not included in the analysis. One adult patient underwent several resections due to an abdominal trauma and received PN before the final SBS diagnosis. This patient was included in the analysis at all time points. One of the pediatric patients died after 410 days of teduglutide treatment due to fulminant sepsis. His death was not related to teduglutide treatment, but was caused by catheter sepsis, as the patient had not yet been weaned from PN. This patient was not included in the analysis at the last assessment date. At the time of the last assessment, one of the adult patients had not reached 12 months on teduglutide treatment (their treatment time was 353 days). This patient was not included in the analysis at the 12-month timepoint.

### 2.3. Variables and Measurement

At the defined times and periods, the following variables were captured. The demographics recorded at diagnosis included anonymized patient ID, sex, date of birth and year of diagnosis, and weight and height (BMI). Clinical characteristics of patients were captured at baseline and at two follow-up times (12 weeks after the index date (with a deviation of ±8 days) and 6 months after the index date (with a deviation of ±21 days), during the last week before the medical check-up. The data collected every week were as follows: intravenous hydration volume, number of intravenous hydration applications, HPN caloric intake, and number of HPN caloric intake applications. Peroral caloric intake per day (including nutritional products), urine output per day, and number of stools per day were calculated as the mean value from the last week before the medical check-up. The BMI was captured on the day of the medical check-up. The weaning-off status was captured at baseline and at four follow-up times: 12 weeks after the index date (with a deviation of ±8 days), 6 months after the index date (with a deviation of ±21 days), 12 months after the index-date, and at the end of the study (4 November 2023). The occurrence of any complications and hospitalizations was recorded if they occurred at any time before the index date.

### 2.4. Sample Size and Bias

Although the studied population was relatively small (n = 16), it represented the general cohort of all patients who met the inclusion criteria, i.e., of all patients with SBS treated with teduglutide in a defined period. One of the adult patients, who started teduglutide treatment one year after the end of PN due to the loss of central venous access sites, was not included in the analysis of changes in recorded parameters over time (however, in the base table of the descriptive description of the general-population patients on teduglutide, this patient is included).

As in other studies that use data from medical records, there is a risk of bias due to missing data (information bias). To avoid this problem, only patients who started treatment within a defined time period that provided a minimum of six months of baseline and who completed a full 6 months of treatment were enrolled. For each parameter, the number of patients with a record is indicated. With regard to comorbidities, confounding factors or interactions that may introduce bias, such as concurrent treatment for a comorbid condition, were not recorded. Hence, these parameters are limited to descriptive analysis of intra-subject changes.

### 2.5. Quantitative Variables and Statistical Analysis

The primary outcomes were analyzed by descriptive statistics. All continuous variables were described using standard statistical measures: number of observations, mean, standard deviation (SD), median, interquartile range (IQR), and minimum and maximum values (range). All categorical variables were summarized as absolute and relative frequencies (percentage).

Secondary outcomes included changes from baseline in disease outcomes during follow-up. For statistical testing of within-individual changes, number of catheter days per week, intravenous hydration volume per week, HPN caloric intake per week, urine output, stool output, and weaning-off status were collected. The measured values for each patient were compared across different time points. The nonparametric Sign test was chosen to test the effect of treatment at the selected time points. To verify the dependence of the HPN status and the duration of teduglutide treatment, the Fisher exact test was used to compare the baseline with another time point. A value of α = 0.05 was taken as statistically significant. Data were processed and analyzed using Microsoft 365 Excel (version 2311), STATISTICA 14 software (version 14.0.0.15) [38].

In the analysis of each parameter, only patients with a record were included.

## 3. Results

### 3.1. Basic Characteristics of the Population

The study included 16 patients treated with teduglutide: 7 children and 9 adults. Their basic characteristics are summarized in Table 1. One of the adult patients started teduglutide treatment 1.8 years after the termination of PN due to the loss of central venous access sites. His basic characteristics are included in the population characteristics in Table 1 but are not included in the analysis of the changes of clinical characteristics due to teduglutide treatment.

### 3.2. Intravenous Hydration

Statistically significant individual changes in intravenous hydration volume were visible after only 12 weeks of teduglutide treatment in the adult population (*p* = 0.0233) (Table 2), and the effect was even more pronounced after 6 months of teduglutide treatment (*p* = 0.0133). There was a median decrease of −2750 mL per week (IQR −3250–(−1750)) after 12 weeks and −4000 mL per week (IQR −6000–(−2750) after 6 months (Table 2). No increase in intravenous hydration volume was observed in any adult patient.

In children, a decrease in intravenous hydration volume per week was visible, but not statistically significant (Table 3). Hydration volume in the pediatric population depends on various factors (such as age), and the results are therefore hard to interpret.

### 3.3. Intravenous Hydration Applications per Week

The reduction in the volume of intravenous hydration in adult patients led to a reduction the in number of intravenous hydration applications per week (Table 2). After 12 weeks, 37.50% of the adult patients had a reduction in the number of hydration applications compared to baseline. In children, most of the patients showed no change in the number of hydration applications after 12 weeks (Table 3). After 6 months, a reduction in the number of hydration applications or complete weaning off hydration was observed in 57.14% of pediatric patients and 75.00% of adult patients, but in children, the effect was not statistically significant (Table 3). No increase in the number of hydration applications was observed in any pediatric or adult patient at 12 weeks or 6 months of treatment.

### 3.4. HPN Caloric Intake

In adult patients, a noticeable decrease in HPN caloric intake was already observed after 12 weeks of treatment. At this time point, only 12.50% of patients had unchanged HPN caloric intake. The HPN caloric intake decreased from a median 13,427.5 kcal per week (IQR 8020–15,890) at baseline to a median 6322.5 kcal per week (IQR 4335–8530) after 12 weeks and a median 3225 kcal per week (IQR 1340–3925) after 6 months (Table 2). This result represents a statistically significant decrease in individual changes in HPN caloric intake (*p* = 0.0233), with median changes of −4710 kcal per week (IQR −7360–(−3610)) after 12 weeks and −9630 kcal per week (IQR −11,832.5–(−4735)) after 6 months (*p* = 0.0133) (Table 2). In summary, all adult patients achieved either a reduction in HPN caloric intake (87.50%) or a complete weaning off HPN caloric intake (12.50%) within 6 months of treatment. No increase in HPN caloric intake was observed in any adult patient (neither at 12 weeks, nor at 6 months of treatment). In children, no statistically significant reduction in HPN caloric intake was observed (Table 3). Caloric intake in pediatric population depends on several factors (such as age or BMI); therefore, it is not appropriate to evaluate the change in energy intake in children.

### 3.5. HPN Caloric Intake Applications per Week

The decrease in HPN caloric intake was associated with a decrease in applications of HPN caloric intake per week. A more pronounced reduction was observed in the adult population. The number of applications decreased from a median of 7 applications per week (IQR 5–7) at baseline to a median of 3.5 applications (IQR 3–6) after 12 weeks and a median of 2.5 applications (IQR 1.5–3) after 6 months (Table 2). This result represents a median decrease of −1 application per week (IQR (−3.5–0) after 12 weeks and a statistically significant decrease after 6 months (*p* = 0.0133), with a median of −3.5 applications per week (IQR −4.5–(−2)) (Table 2). After 12 weeks, the number of HPN applications remained unchanged in only 50% of patients, and all adult patients reduced the number of HPN applications or weaned off HPN within 6 months of treatment. None of the adult patients showed an increase in the number of HPN applications after either 12 weeks or 6 months of treatment. In children, the decrease in HPN caloric intake applications was not significant (Table 3). A reduction was achieved in the same proportion (28.57%) of pediatric patients in both time periods. After 12 weeks of treatment, an increase in the number of HPN applications was observed for one pediatric patient (14.29%). After 6 months, the number of HPN applications for the patient in question decreased again to the baseline level.

### 3.6. Peroral Caloric Intake (Kcal per Day)

In addition to caloric intake from HPN, all SBS patients treated with teduglutide also had peroral (p.o.) food intake (results not shown). At baseline, 46.67% of all patients had a normal p.o. caloric intake, 40.00% had a lower p.o. caloric intake and 13.33% a higher p.o. caloric intake. After 12 weeks, the share of normal p.o. intake increased at the expense of both lower and higher p.o. intake. After 6 months, 60.00% of patients reached normal p.o. caloric intake. In adult patients, after 12 weeks of treatment, all patients with lower p.o. caloric intake than normal had already reached the normal level. After 6 months of treatment, all adult patients had a normal p.o. caloric intake. In children, only 1 patient had a normal p.o. caloric intake after 6 months of treatment. After 12 weeks, the p.o. caloric intake increased in 57.14% of the pediatric patients, but after 6 months, a decrease in p.o. caloric intake to a two-thirds level was observed for 2 patients (28.57%).

### 3.7. BMI

Both increases and decreases were observed in the pediatric population. After 12 weeks and 6 months of treatment, there was an increase in BMI in 57.14% of patients and a decrease in 42.86% of patients compared to baseline. In adults, after 6 months, an increase in BMI was observed in 75.00% of patients. None of these results was statistically significant (Table 2 and Table 3).

### 3.8. Urine Output

An increase in urine output in adults was observed after only 12 weeks of treatment (62.50% of patients). After 6 months of treatment, all adult patients reported increased urine output. There was a median increase of 160 mL per day (IQR 0–350) after 12 weeks and a statistically significant increase (*p* = 0.0133) after 6 months, with a median increase of 575 mL per day (IQR 300–645) (Table 2). In the pediatric population, measuring patient diuresis is difficult because most of the patients use diapers. For this reason, the daily volume of urine output was known only in n = 2 patients and a statistical evaluation of the results was not possible (Table 3).

### 3.9. Stool Output

The change in stool output can be evaluated in patients who had the same type of stool at all time points, i.e., patients with normal stool (patients with a record n = 9). Patients with stomas (n = 6) were excluded from the analysis. A more pronounced reduction in the daily stool output was observed in the adult population. All adult patients with a record (n = 4) experienced a reduction in stool output that was not statistically significant (Table 2). In children (n = 5), all patients showed a reduction in stool output after 12 weeks, but after 6 months of treatment, 40.00% of patients returned to the baseline level (Table 3).

### 3.10. Weaning off HPN

Weaning off HPN was evaluated at 4 timepoints: 12 weeks after baseline, 6 months after baseline, 12 months after baseline and at the day of the last assessment (4 November 2023) (Figure 1). The median time from baseline to the date of the last assessment was 2.33 years (IQR 1.19–2.90): 2.72 years (IQR 2.33–3.07) in children and 2.09 years (IQR 1.04–2.26) in adults).

**Figure 2:**
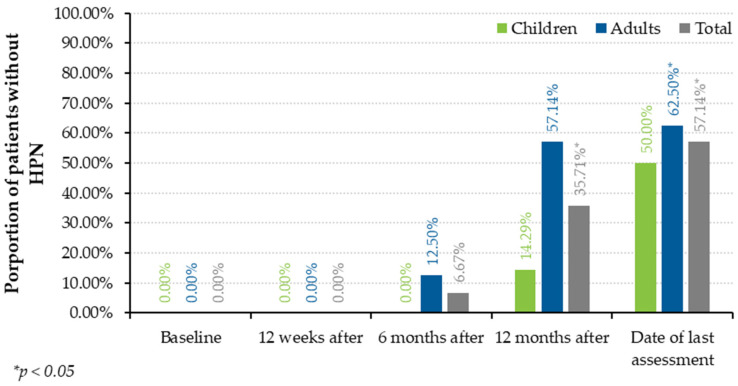
Proportion of patients weaned off HPN.

At the 12-week timepoint, no patient had weaned off HPN. The first patient to wean off HPN did so within 6 months of treatment (adult; 92 days of treatment). Within one year of teduglutide treatment, 35.71% of patients had weaned off HPN (1 child, 14.29%; 4 adults, 57.14%). By the end of the study, 50.00% of children (3/6) and 62.50% of adults (n = 5/8; *p* = 0.0256) had weaned off HPN after a median of 1.07 (IQR 0.92–1.07) and 0.98 (IQR 0.76–1.00) years, respectively. In summary, 8 patients (n = 14; 57.14%; *p* = 0.0019) weaned off HPN after a median of 0.99 years (IQR 0.84–1.07) (Figure 2, Table 4).

## 4. Discussion

Although there have been several studies on teduglutide efficacy in SBS patients, this study is the first to report real-world data on teduglutide-treated SBS patients in the Slovak Republic. Additionally, to the best of our knowledge, no other study has enabled the comparison of the effects of teduglutide treatment between the adult and pediatric populations. Our results showed that although teduglutide had similar effects in both populations, in children, the results were less pronounced, with most not being statistically significant, whereas in adults, there were statistically significant changes in all clinical characteristics except for BMI and number of stools per day.

A metanalysis of 10 studies (2 RTCs and 8 observational) [39] estimated a weaning-off rate of 11% at 6 months, 17% at one year, and 21% at two years or more. In our study, the weaning-off rate was 6.67% at six months and 35.71% at one year for the whole population; it was 12.50% at 6 months and 57.14% at one year for the adults alone. Our results at 12 months clearly exceed these estimations, but the weaning-off rates found in the selected studies are very heterogenous, ranging from 0% [40,41] to 41% [42] at six months and from 9% [43] to 29% [44] at one year. In a real-world observational cohort study by Joly et al. [45], 24% of patients weaned off HPN within 24 weeks of treatment. In our study, only one patient was able to wean off before the six-month timepoint. In the adult population, the median time from baseline to weaning off HPN was 0.98 years (IQR 0.76–1), a finding in accordance with the results of the analysis of the achievement of enteral autonomy in the STEPS trials, where all patients who achieved enteral autonomy required ≥6 months of teduglutide treatment [32]. In a post hoc analysis of adult patients treated with teduglutide in five clinical trials and their extension studies, the patients achieved enteral autonomy after a median of 1.7 years (89 weeks), whereas most of these patients (12 of 16) weaned off HPN after ≥1 year of teduglutide treatment [46]. By contrast, in the single-center, retrospective study by Puello et al. [47], among the five patients who were able to wean off HPN (of 18 total; 27.78%), three patients weaned off after three months of teduglutide therapy, while 1 patient weaned off HPN within six months and the last weaned off HPN after 14 months of treatment.

Among the pediatric patients, 50.00% (n = 3) were able to wean off HPN after a median of 1.07 years (IQR 0.92–1.07). The first patient to be able to do so weaned off HPN within 12 months of treatment. An analysis of 14 studies performed in pediatric patients revealed that a total of 36 out of 223 patients (16.14%) achieved enteral autonomy after a median of 24 weeks of treatment [48]. The results of the studies included were again heterogenous. For example, in the real-world study by Ramos Boluda et al. [49], 70.59% (12/17) of patients was able to wean off (three after three months, four at six months, five after twelve months), whereas in the single-center study by Kinberg et al. [50], none of the patient treated with teduglutide for 3–12 months were able to wean off HPN. In the analysis of two open-label phase 3 studies and one extension study, two children receiving teduglutide achieved enteral autonomy after 12 weeks and 28 weeks of treatment, respectively [51].

The statistically significant decrease in intravenous hydration intake and HPN caloric intake in the adult population after teduglutide treatment confirmed the positive effect of teduglutide on HPN dependence. A significant decrease in both characteristics was observed after only 12 weeks of treatment. After six months of treatment, a reduction in the weekly hydration volume or a complete weaning off was observed in all adult patients. Likewise, in a retrospective study comprising 18 SBS patients treated with teduglutide, a decrease in HPN caloric intake was observed after only three months of treatment [47]. The patients in question reached a mean reduction in HPN caloric uptake of −2078.9 kcal per week after three months and −2524.8 kcal per week after six months of teduglutide treatment. Those numbers are much lower than the mean reductions in the patients in this study: −5027.5 kcal per week and −8870.63 kcal per week, respectively (Table 2). In another study, a mean decrease in HPN caloric uptake of −3052 kcal per week was observed after 24 weeks [45].

In children, we observed decreases in HPN caloric intake median of −2.78% after 12 weeks and −33.95% after 6 months, but this result was not statistically significant. Nevertheless, in a study on infants (4–12 months of age) and children (1–15 years of age) at week 24, the PS caloric intake had reduced by −33.4 ± 17.8% for infants and −35.2 ± 33.7% for the children [51]. In a 24-week phase III randomized, double-blind trial, a reduction in HPN caloric intake was also observed [52].

Another of the effects of teduglutide treatment was increased urine output in the adult population. After six months, the urine production increased by a median of 575 mL per day. A similar effect was observed in a randomized placebo-controlled trial, wherein patients receiving teduglutide produced significantly more urine (+367 mL per day) at week 24 compared to baseline [53].

Our findings are in line with previously published results of real-world-data studies. We confirmed the efficacy of teduglutide in reducing HPN dependence, as observed through the decreases in the volume of intravenous hydration, number of intravenous hydration applications per week, HPN caloric intake, and HPN applications per week and in complete weaning off HPN in 57.14% of the patients. In summary, all adult patients reached either a reduction in HPN caloric intake or a complete weaning off HPM within six months of treatment.

Our analysis had some limitations. We did not consider other possible factors that could influence weaning-off status, like the differences in residual length and the absorption ability of the rest of the small intestine. Other limitations include the different kinds of HPN bags used by the patients, which do not enable a comparison of the total HPN volume reduction between the patients. Although we had a general cohort of teduglutide-treated SBS patients in Slovak Republic, we had to exclude one patient, who started teduglutide treatment 1.8 years after the end of PN due to the loss of central venous access sites. The retrospective design does not enable the collection of additional information from patients.

## 5. Conclusions

Teduglutide treatment in SBS patients leads to considerable reduction in the use of HPN or even weaning off HPN in both children and adult patients.

Statistical analysis of responses in the pediatric population failed to demonstrate any effect of teduglutide on the observed characteristics. Statistical analysis of responses in the adult population confirmed the statistical significance of the effect of teduglutide on intravenous hydration volume, number of intravenous hydration applications per week, HPN caloric intake, number of HPN caloric applications per week, urine output, and weaning off HPN.

## Figures and Tables

**Table 1:** Basic characteristics of the patients.

Patient Characteristics	Children (n = 7)	Adults (n = 9)	Total (n = 16)
Male, n (%)	5 (71.43%)	5 (55.56%)	10 (62.5%)
Female, n (%)	2 (28.57%)	4 (44.44%)	6 (37.5%)
Age at diagnosis (years)			
Mean (SD)	0.13 (0.25)	43.52 (15.09)	24.54 (24.81)
Median (IQR)	0.04 (0.01–0.13)	37.19 (30.32–54.69)	29.43 (0.04–46.17)
Range	0–0.69	29.01–72.16	0–72.16
Type of SBS			
Type I, n (%)	1 (14.29%)	3 (33.33%)	4 (25%)
Type II, n (%)	3 (42.86%)	3 (33.33%)	6 (37.5%)
Type III, n (%)	3 (42.86%)	2 (22.22%)	5 (31.25%)
Other, n (%) ^1^	0 (0%)	1 (11.11%)	1 (6.25%)
Underlying diagnosis			
Crohn disease	0 (0%)	4 (44.44%)	4 (25%)
Volvulus	3 (42.86%)	0 (0%)	3 (18.75%)
Mesenteric thrombosis	0 (0%)	2 (22.22%)	2 (12.5%)
Ileus	0 (0%)	2 (22.22%)	2 (12.5%)
Necrotizing enterocolitis	1 (14.29%)	0 (0%)	1 (6.25%)
Intestinal atresia	1 (14.29%)	0 (0%)	1 (6.25%)
Gastroschisis, intestinal atresia	1 (14.29%)	0 (0%)	1 (6.25%)
Malabsorption after surgical procedure	0 (0%)	1 (11.11%)	1 (6.25%)
Gastroschisis	1 (14.29%)	0 (0%)	1 (6.25%)
Remaining length of small intestine (cm)			
Patients with record, n ^2^	n = 6	n = 7	n = 13
Mean (SD)	25 (18.83)	57.14 (18.9)	42.31 (24.58)
Median (IQR)	19.5 (15–20)	60 (40–80)	40 (19.5–60)
Range	6–60	30–80	6–80
Age at first HPN administration (years)			
Mean (SD)	1.18 (0.95)	43.58 (15.19)	25.03 (24.4)
Median (IQR)	1.1 (0.55–1.39)	37.3 (30.3–54.73)	29.42 (1.11–46.16)
Range	0.04–3.09	29.01–72.57	0.04–72.57
Time from the first HPN administration to the baseline (years)			
Mean (SD)	5.85 (4.18)	3.81 (3.57)	4.7 (3.86)
Median (IQR)	6.89 (1.71–8.44)	3.51 (0.99–5.61)	3.69 (1.23–7.78)
Range	1.22–12.27	0.72–11.72	0.72–12.27
Number of hospitalizations before teduglutide treatment (per patient)			
Patients with record, n ^3^	n = 6	n = 9	n = 15
Mean (SD)	12 (5.87)	2.78 (3.35)	6.47 (6.37)
Median (IQR)	13.5 (7.25–16)	3 (0–4.5)	4 (0–13)
Range	2–19	0–10	0–19
Number of complications before teduglutide treatment (per patient), (does not include hospitalizations)			
Patients with record, n ^3^	n = 6	n = 9	n = 15
Mean (SD)	0.17 (0.41)	1.33 (1.66)	0.87 (1.41)
Median (IQR)	0 (0–0.25)	1 (0–2)	0 (0–2)
Range	0–1	0–5	0–5
Age at baseline (years)			
Mean (SD)	7.03 (4.71)	47.39 (14.03)	29.73 (23.27)
Median (IQR)	7.99 (2.26–11.53)	42.49 (37.15–57.64)	32.94 (8.25–49.17)
Range	1.26–13.39	29.93–73.82	1.26–73.82

^1^ Colectomy and repeated short resections of the small intestine. ^2^ In 3 patients (1 child, 2 adults), the remaining length of small intestine was not known. ^3^ One of the pediatric patients was not included due to continuous hospitalization since birth.

**Table 2:** Adults (n = 8): HPN days per week, intravenous hydration (mL/week), HPN caloric intake (kcal/week), peroral caloric intake (kcal/day), including nutritional products, BMI, urine output (mL/day), stools per week. Individual changes in HPN days per week, intravenous hydration (mL/week), HPN caloric intake (kcal/week), peroral caloric intake (kcal/day), including nutritional products, BMI, urine output (mL/day), stools per week; baseline vs. the 12-week and 6-month follow-up times.

Characteristics	Baseline	12 Weeks after	6 Months after	12 Weeks after vs. Baseline	6 Months after vs. Baseline
Intravenous hydration (mL/week)					
Patients with record, n ^1^	8	8	8	8	8
Mean (SD)	7875 (3870.68)	5500 (4440.08)	3437.5 (2367)	−2375 (1246.42)	−4437.5 (2145.39)
Median (IQR)	6500 (5000–10,500)	3250 (2500–8250)	2750 (2000–5500)	−2750 ((−3250)–(−1750))	−4000 ((−6000)–(−2750))
Range	5000–14,000	2000–14,000	0–7000	(−3500)–(0)	(−8000)–(−2000)
*p*-value (Sign test)	-	-	-	**0.0233**	**0.0133**
Intravenous hydration applications per week					
Patients with record, n ^1^	8	8	8	8	8
Mean (SD)	6.13 (0.99)	5.5 (1.51)	4.25 (2.19)	−0.63 (1.06)	−1.88 (1.64)
Median (IQR)	6.5 (5–7)	5.5 (4.5–7)	5 (3–5.5)	0 ((−1)–(0))	−2 ((−2.5)–(−0.5))
Range	5–7	3–7	0–7	(−3)–(0)	(−5)–(0)
*p*-value (Sign test)	-	-	-	0.2482	**0.0412**
HPN caloric intake (kcal/week)					
Patients with record, n ^1^	8	8	8	8	8
Mean (SD)	11,705.63 (4916.73)	6678.13 (2964.8)	2835 (1915.52)	−5027.5 (2845.59)	−8870.63 (4522.33)
Median (IQR)	13,427.5 (8020–15890)	6322.5 (4335–8530)	3225 (1340–3925)	−4710 ((−7360)–(−3610))	−9630 ((−11,832.5)–(−4735))
Range	3080–15,890	3080–11,970	0–5700	(−8860)–(0)	(−15,890)–(−2680)
*p*-value (Sign test)	-	-	-	**0.0233**	**0.0133**
HPN caloric intake, applications per week					
Patients with record, n ^1^	8	8	8	8	8
Mean (SD)	5.88 (1.81)	4.25 (1.91)	2.38 (1.51)	−1.63 (1.85)	−3.5 (1.93)
Median (IQR)	7 (5–7)	3.5 (3–6)	2.5 (1.5–3)	−1 ((−3.5)–(0))	−3.5 ((−4.5)–(−2))
Range	2–7	2–7	0–5	(−4)–(0)	(−7)–(−1)
*p*-value (Sign test)	-	-	-	0.1336	**0.0133**
BMI					
Patients with record, n ^1^	8	8	8	8	8
Mean (SD)	21.81 (3.25)	21.71 (2.97)	22.74 (2.8)	−0.1 (2.54)	0.93 (1.72)
Median (IQR)	23.23 (19.2–24.16)	20.02 (19.65–24.85)	22.33 (20.93–24.7)	0.68 ((−1.94)–(1.77))	0.58 ((−0.1)–(2.35))
Range	16.38–24.91	18.67–25.95	18.67–27.34	(−4.57)–(2.73)	(−1.73)–(3.52)
*p*-value (Sign test)	-	-	-	0.7237	0.2888
Urine output (mL/day)					
Patients with record, n ^1^	8	8	8	8	8
Mean (SD)	922.5 (315.76)	1130 (380.68)	1415 (344.26)	207.5 (229.77)	492.5 (202.89)
Median (IQR)	900 (650–1080)	1000 (950–1320)	1250 (1200–1575)	160 ((0)–(350))	575 ((300)–(645))
Range	600–1520	700–1800	1200–2070	(0)–(640)	(200)–(700)
*p*-value (Sign test)	-	-	-	0.0736	**0.0133**
Stools per day					
Patients with record, n ^1,2^	4	4	4	4	4
Mean (SD)	9 (1.15)	6 (2.16)	2.75 (1.5)	−3 (1.83)	−6.25 (1.26)
Median (IQR)	9 (8–10)	5.5 (4.5–7.5)	2 (2–3.5)	−3 ((−4.5)–(−1.5))	−6 ((−7)–(−5.5))
Range	8–10	4–9	2–5	(−5)–(−1)	(−8)–(−5)
*p*-value (Sign test)	-	-	-	0.1336	0.1336

^1^ One of the adult patients, who started teduglutide treatment 1.8 years after the end of PN due to the loss of central venous access sites is not included. ^2^ Only patients with records for this parameter were included in the analysis. Patients with stomas were not included. Statistically significant *p* -values α < 0.05 are highlighted in bold.

**Table 3:** HPN days per week, intravenous hydration (mL/week), HPN caloric intake (kcal/week), peroral caloric intake (kcal/day), including nutritional products, BMI, urine output (mL/day), stools per week. Individual changes in HPN days per week, intravenous hydration (mL/week), HPN caloric intake (kcal/week), peroral caloric intake (kcal/day), including nutritional products, BMI, urine output (mL/day), stools per week; baseline vs. the 12-week and 6-month follow-up times.

Characteristics	Baseline	12 Weeks after	6 Months after	12 Weeks after vs. Baseline	6 Months after vs. Baseline
Intravenous hydration (mL/week)					
Patients with record, n ^1^	4	4	4	4	4
Mean (SD)	2562.5 (2995.66)	1962.5 (2534.22)	1640 (3109.08)	−600 (617.79)	−922.5 (563.05)
Median (IQR)	1250 (750–4375)	1125 (250–3675)	130 (0–3280)	−500 ((−1075)–(−125))	−725 ((−1250)–(−595))
Range	750–7000	0–5600	0–6300	(−1400)–(0)	(−1750)–(−490)
*p*-value (Sign test)	-	-	-	0.2482	0.1336
Intravenous hydration applications per week					
Patients with record, n ^1^	7	7	7	7	7
Mean (SD)	5.86 (1.95)	4.86 (2.91)	3.86 (3.24)	−1 (1.41)	−2 (2.58)
Median (IQR)	7 (3–7)	7 (2–7)	4 (0–7)	0 ((−3)–(0))	−1 ((−3)–(0))
Range	3–7	0–7	0–7	(−3)–(0)	(−7)–(0)
*p*-value (Sign test)	-	-	-	0.2482	0.1336
HPN caloric intake (kcal/week)					
Patients with record, n	7	7	7	7	7
Mean (SD)	4524.29 (2594.38)	3950 (2697.38)	3314.14 (2358.64)	−574.29 (1551.92)	−1210.14 (1934.18)
Median (IQR)	4200 (1800–7000)	4900 (1600–6300)	3304 (1400–4550)	−50 ((−1300)–(700))	−900 ((−1925)–(350))
Range	1800–8400	500–7700	900–7700	(−3500)–(700)	(−5096)–(700)
*p*-value (Sign test)	-	-	-	1.0000	0.4497
HPN caloric intake, applications per week					
Patients with record, n	7	7	7	7	7
Mean (SD)	6.14 (1.46)	5.57 (1.99)	5.43 (2.07)	−0.57 (1.4)	−0.71 (1.25)
Median (IQR)	7 (4–7)	7 (4–7)	7 (4–7)	0 ((−2)–(0))	0 ((−2)–(0))
Range	4–7	2–7	2–7	(−3)–(1)	(−3)–(0)
*p*-value (Sign test)	-	-	-	1.0000	0.4795
BMI					
Patients with record, n	7	7	7	7	7
Mean (SD)	14.13 (1.66)	14.14 (1.45)	13.85 (1.94)	0 (1.11)	−0.28 (1.23)
Median (IQR)	14.06 (13.64–15.5)	14.59 (13–15.02)	14.47 (13.58–15,19)	0.37 ((−0.51)–(0.9))	−0.42 ((−1.22)–(0.99))
Range	10.84–15.61	11.22–15.5	9.67–15.35	(−2.2)–(0.96)	(−1.92)–(1.28)
*p*-value (Sign test)	-	-	-	1.0000	1.0000
Urine output (mL/day)					
Patients with record, n ^2^	2	2	2	2	2
Mean (SD)	625 (35.36)	625 (35.36)	440 (296.98)	0 (70.71)	−185 (261.63)
Median (IQR)	625 (600–650)	625 (600–650)	440 (230–650)	0 ((−50)–(50))	−185 ((−370)–(0))
Range	600–650	600–650	230–650	(−50)–(50)	(−370)–(0)
*p*-value (Sign test)	-	-	-	x	x
Stools per day					
Patients with record, n ^3^	5	5	5	5	5
Mean (SD)	8.8 (3.35)	6.8 (2.17)	6.6 (4.34)	−2 (1.22)	−2.2 (2.28)
Median (IQR)	8 (6–10)	6 (5–8)	6 (4–6)	−2 ((−2)–(−1))	−2 ((−4)–(0))
Range	6–14	5–10	3–14	(−4)–(−1)	(−5)–(0)
*p*-value (Sign test)	-	-	-	0.0736	0.2482

^1^ 3 pediatric patients did not have separate hydration but received hydration through the so-called “all in one” bags as part of HPN. In these 3 patients, the intravenous hydration volume is not known; therefore, they were not included in the analysis of changes in intravenous hydration. The number of applications of intravenous hydration in these patients is equal to the number of applications of HPN caloric intake. ^2^ 5 patients had no record for this parameter because most of the patients use diapers and were not included in the analysis. ^3^ Only patients with a record for this parameter were included in the analysis. Patients with stomas were not included. x indicates that the *p*-value could not be calculated.

**Table 4:** Number of patients, their age at HPN termination and time from baseline to weaning off.

Weaning Off	Children (n = 7)	Adults (n = 9)	Total (n = 16)
Weaning off HPN after teduglutide treatment initiation, n (%) ^1^	3 (50.00%)	5 (62.5%)	8 (57.14%)
Age at termination of HPN (years)			
Patients with record, n ^1^	n = 3	n = 5	n = 8
Mean (SD)	5.67 (3.04)	53.71 (15.9)	35.69 (27.66)
Median (IQR)	4.78 (x)	53.75 (38.35–69.05)	38.35 (5.85–60.89)
Range	3.18–9.06	37.19–74.82	3.18–74.82
Time from baseline to weaning off (years)			
Patients with record, n ^1^	n = 3	n = 5	n = 8
Mean (SD)	1.02 (0.09)	0.85 (0.38)	0.91 (0.3)
Median (IQR)	1.07 (0.92–1.07)	0.98 (0.76–1.00)	0.99 (0.8–1.07)
Range	0.92–1.07	0.25–1.25	0.25–1.25

^1^ One of the adult patients, who started teduglutide treatment 1.8 years after the end of PN due to the loss of central venous access sites, is not included. x indicates that the IQR could not be calculated.

## Data Availability

Data is unavailable due to privacy or ethical restrictions. L.G. has full access to all the data in the study and takes responsibility for the integrity of the data and the accuracy of the data analysis. Data can be obtained from the corresponding author L.G.
